# Cross-sectional and longitudinal associations between the built environment and walking: effect modification by socioeconomic status

**DOI:** 10.1186/s12889-022-13611-0

**Published:** 2022-06-21

**Authors:** Chelsea D. Christie, Christine M. Friedenreich, Jennifer E. Vena, Liam Turley, Gavin R. McCormack

**Affiliations:** 1 Department of Community Health Sciences, Cumming School of Medicine, University of Calgary, TRW 3rd floor, 3280 Hospital Drive NW, Calgary, Alberta T2N 4Z6 Canada; 2 Department of Cancer Epidemiology and Prevention Research, Cancer Care Alberta, Alberta Health Services, 2210 2nd St SW, Calgary, Alberta T2S 3C3 Canada; 3 Alberta’s Tomorrow Project, Cancer Care Alberta, Alberta Health Services, Richmond Road Diagnostic and Treatment Centre, 1820 Richmond Road SW, Calgary, Alberta T2T 5C7 Canada; 4 School of Architecture, Planning and Landscape, University of Calgary, 2500 University Dr NW, Calgary, Alberta T2N 4N1 Canada

**Keywords:** Built environment, Physical activity, Alberta’s tomorrow project, Socioeconomic status, Walking

## Abstract

**Background:**

Although socioeconomic status (SES) has been shown to modify associations between the neighborhood built environment and physical activity, contradictory results exist. Objectives of this cross-sectional and longitudinal analysis were to: 1) examine whether overall neighborhood walkability and specific built characteristics were associated with walking among adults at a single point in time and after they relocate neighborhoods, and 2) test for effect modification of these associations by SES.

**Methods:**

We linked longitudinal data from 703 adults who relocated urban neighborhoods between two waves of Alberta’s Tomorrow Project (2008–2015) to neighborhood built environment data. We created a walkability index from measures of population counts, street connectivity, and destination diversity within 400 m of participants’ homes. In cross-sectional analyses, we used generalized linear models to estimate associations between built characteristics and minutes walked per week at baseline. For the longitudinal analyses, we used fixed-effects linear regression models to estimate associations between changes in built characteristics and minutes walked per week. We also assessed if indicators of SES (individual education or household income) modified both sets of associations.

**Results:**

Most cross-sectional and longitudinal associations were small and statistically non-significant. Neighborhood population count (*b* = 0.03, 95% CI: 0.01, 0.07) and street connectivity (*b* = − 1.75, 95% CI: − 3.26, − 0.24) were cross-sectionally associated with walking duration among the overall sample. None of the longitudinal associations were statistically significant among the overall sample. There was limited evidence of effect modification by SES, however, we found negative cross-sectional associations between street connectivity and walking among adults with lower education and income, and a positive association between percent change in walkability and change in walking among lower educated adults.

**Conclusions:**

Despite population count and street connectivity being associated with walking at baseline, changes in these built environment variables were not associated with changes in walking following residential relocation. Our findings also provide evidence, albeit weak, that changes in neighborhood walkability, resulting from residential relocation, might more strongly affect walking among low SES adults. Further longitudinal research is needed to examine built environment characteristics with walking for different purposes and to test for inequitable socioeconomic impacts.

**Supplementary Information:**

The online version contains supplementary material available at 10.1186/s12889-022-13611-0.

## Background

Regular physical activity can reduce the risk of chronic disease and premature mortality [1–3]. The World Health Organization’s Global Action Plan for Physical Activity and Health has a target of a 15% reduction in inactivity levels by 2030 and recommends several policy actions, including the creation of physical activity supportive environments [4]. Evidence, derived mainly from cross-sectional studies, suggests that numerous neighborhood built characteristics, such as street connectivity, land-use mix, residential density, and walkability (the combination of several built characteristics) are associated with physical activity [5–7]. However, cross-sectional studies are limited in their ability to establish temporality in the associations between built environment characteristics and walking [7, 8]. Furthermore, it is possible that participants self-select into neighborhoods with particular built characteristics based on their walking preferences leading to biased estimates in cross-sectional studies [9, 10]. Longitudinal studies, and specifically those examining the impacts of residential relocation, are able to measure physical activity before and after participants have been exposed to a new neighborhood built environment [11, 12]. Several residential relocation studies have found that increases in neighborhood walkability are associated with increases in walking duration among adults [13, 14]; however, the associations between specific built characteristics, such as street connectivity, land-use mix, and availability of public transportation, with walking are mixed [15, 16].

Despite evidence suggesting positive associations between socioeconomic status (SES) and physical activity [17], there is inconsistency regarding potential benefits of the built environment for physical activity among socioeconomically disadvantaged groups [18–20]. A systematic review of longitudinal studies investigating associations between the built environment and physical activity [19] found that most studies reported no evidence of effect modification by individual-level SES, except for one study, which found that new walking and cycling routes were used more by people with higher incomes, higher education levels, and by people who were currently employed [21]. In contrast, in their narrative review, Adkins et al. [20] reported that most studies in their review found weaker associations between the built environment and physical activity among socioeconomically disadvantaged groups compared to socioeconomically advantaged groups. Given the limited number of studies and mixed findings on differential impacts of the built environment on walking for different SES groups, more research is needed.

The objectives of this study were to: 1) examine whether or not overall neighborhood walkability and specific built characteristics were associated with walking among adults at a single point in time (cross-sectional) and after they relocate neighborhoods (longitudinal), and 2) test for effect modification of these associations by SES (education and household income).

## Methods

### Study and sample design

This analysis used data from Alberta’s Tomorrow Project (ATP), a prospective cohort study designed to investigate the etiology of cancer and other non-communicable diseases [22]. ATP recruited adults aged 35–69 years living in Alberta, Canada who had no history of cancer, other than non-melanoma skin cancer [22], between 2000 and 2008 using random digit dialing [23]. Of the 63,486 eligible and interested adults contacted, 31,072 consented to participate [23]. Participants completed a survey at the time of enrollment to measure demographic, personal and family health, and lifestyle information, and then follow-up surveys in 2008 and 2009–2015 for updating and collecting new information. The flow of sample recruitment and participation through ATP over time have been described elsewhere [22].

For this analysis, we focused on two time points using the surveys released in 2008 (referred to herein as ‘baseline’) and in 2009–2015 (referred to as ‘follow-up’) due to the consistency of the physical activity tool used in both surveys (described below). The eligibility criteria for this study included participants who completed the baseline and follow-up surveys and those who relocated (both to and from an urban area) between the two time points (based on a change in residential postal code). Those participants are hereafter referred to as “movers” for simplicity. Note that we retained “non-movers” (those who completed both the baseline and follow-up surveys and stayed in an urban area between the two time points) to test for differences between “non-movers” and “movers” before excluding them from the sample. Among the 9779 urban-dwelling participants who completed both baseline and follow-up surveys, participants were excluded if they did not move (*n* = 8952), had missing data (*n* = 123), or were immobile at baseline (*n* = 1; assessed through the mobility-related component of the EQ-5D [24]), resulting in a sample size for our analysis of 703 participants. The University of Calgary Conjoint Research Ethics Board approved this study (REB17–1466).

### Measures

#### Exposure variables

The neighborhood built environment exposure measures for this analysis consisted of specific built environment characteristics (population counts, diversity of destinations, and street connectivity) and a composite walkability index estimated for 400-m Euclidean buffers around each participant’s residential 6-digit postal code at baseline and follow-up. In Canada, urban residential postal codes have a geographical area that typically ranges from one apartment building to one city block [25], and postal codes can be used as a valid proxy for household locations [26]. A 400-m buffer was chosen because it represents the approximate distance that can be travelled by walking in approximately 5 minutes [27]. Geographic Information Systems (GIS) were used to process built environment data for all urban postal codes in Alberta for each year between 2008 and 2015, and participants’ home locations were linked to objective built environment data with their postal code for baseline and follow-up based on the year they completed each survey.

Population counts were estimated using 2006, 2011, and 2016 Statistics Canada Census data. Population counts for non-census years were estimated based on averages imputed from the available census data. Diversity of destinations was estimated by summing the number of different destination types (classified by Standard Industry Codes obtained from DMTI Spatial’s CanMap Enhanced Points of Interest data). Examples of destination types included hardware stores, department stores, grocery stores, restaurants, banks, libraries, barbershops, museums, and schools. Street connectivity was estimated by the count of three and four-way street intersections within the buffer (derived from DMTI Spatial’s CanMap Streetfiles and Route Logistics dataset). For the street connectivity measure, we summed the number of three- and four-way street intersections together, but with three-way intersections weighted half as strongly as four-way intersections. These specific built characteristics have been used in a previous study with ATP data that found associations between the built environment and weight status [28].

The walkability index was created using the built characteristics described above (i.e., population counts, diversity of destinations, and connectivity). For the walkability index, the built environment characteristics were standardized relative to the maximum value of each characteristic observed between 2008 and 2015 across all urban postal codes in Alberta (making the scores relative to urban walkability values across the province). The maximum values found for each built characteristic were: 1) population count = 7956; 2) number of different destination types = 23; 3) number of four-way intersections = 51, and; 4) number of three-way intersections = 92. This percent of maximum possible (“POMP”) method [29, 30] results in a standardized score for each built characteristic that is a proportional score bound by 0 and 100%. The four standardized scores were summed together. Similar to prior research that down-weighted three-way intersections relative to four-way intersections [31], and consistent with our connectivity measure, the three-way intersections variable in our index was given a weight of 0.5 instead of one because three-way intersections have been shown to have a weaker association with walking compared to four-way intersections [32]. The resulting walkability index was bounded at 0, with a theoretical maximum of 350 (see Supplementary Material for more detailed information about the built environment measures).

After estimating walkability values at both time points, changes in the walkability values were operationalized in three different ways: 1) per 10-unit change; 2) percent change; and 3) per tertile of change (corresponding to decreased walkability, minimal change, and increased walkability).

To assess convergent validity, we estimated a Pearson correlation coefficient comparing the walkability index for 2012 with an external measure, the 2012 Walk Score®, a publicly available walkability index that reflects the level of access to nearby walkable amenities. Specifically, Walk Score® estimates neighborhood walkability based on proximity to amenities (e.g., grocery stores, restaurants, parks, libraries). Amenities within a five-minute walking distance are given the most points and a decay function is used to provide fewer points, up to a 30-minute walk. The Walk Score® algorithm has been shown to be a valid indicator of neighborhood walkability [33, 34] and is positively associated with walking [35], including among adults with low incomes [36, 37]. Our walkability index was positively correlated with Walk Score® (*r* = 0.69, *p* < 0.001; data not shown).

#### Walking

Both surveys measured walking during the past week using the International Physical Activity Questionnaire (IPAQ) short or long forms, which have been validated in multiple countries, including Canada [38]. The IPAQ asks participants to report on how many days they walked (for at least 10 minutes) during the previous week and how much time they usually spent walking on one of those days. Walking minutes per week at each time point was estimated following the IPAQ scoring system [38] and change scores were calculated to estimate changes in walking minutes per week from baseline to follow-up.

#### SES potential effect modifiers

The two potential effect modifiers, baseline education (≤ high school versus > high school) and baseline household income (low-income household versus not a low-income household) were examined. Participants were asked to report the highest level of education they finished, with nine response options ranging from “did not complete grade eight” to “completed university post-graduate degree”. For the income question, participants were asked to report their total household income before taxes, with 18 response options ranging from “less than $10,000 to “$250,000 or more”. The 2006 Statistics Canada Census data were used to determine the household low-income status cut point. Participants who reported a household income below the 2006 median income level for Alberta ($63,988) were classified as living in a household with a lower income [39]. Due to the categories used in collecting the data, $60,000 was used as the cut point. Two alternative measures of household income were also considered as potential effect modifiers for both the cross-sectional and longitudinal sets of analyses: 1) tertiles of baseline income, and 2) the Statistics Canada Market Basket Measure (a low-income line threshold, which reflects differences in living costs across regions and across household sizes in Canada). Tertiles of baseline income were created from using the midpoint of each income response category. We averaged the Market Basket Measure for all urban Alberta regions and then calculated separate cut points for each year and household size using the square root equivalency recommended by Statistics Canada [40].

#### Covariates

Data on age, sex, relationship status (married/common law, single/never married), IPAQ survey type (short or long-form), and years between surveys were available from self-administered questionnaires. Baseline age, sex, relationship status, the presence of children at home, season of survey completion, and years between surveys were included in the cross-sectional models. Changes in relationship status, presence of children at home, season of survey completion, as well as follow-up IPAQ survey type (short or long form) and years between surveys were included in the longitudinal models. We selected covariates based on their known associations with walking, their availability within the data, and/or whether they could potentially explain relocation to higher or lower levels of walkability (e.g., change in the presence of children in the home [41]).

### Statistical analyses

Descriptive analyses compared movers and non-movers on baseline demographic characteristics, after which non-movers were excluded from the remaining analysis. We used independent samples t-tests and Pearson’s chi-square tests, as appropriate, to test for differences between non-movers and movers. Similarly, baseline demographic characteristics for participants from the three walkability-change groups (tertiles of walkability-change) were compared using one-way analysis of variance (ANOVA) and Pearson’s chi-square tests.

For the cross-sectional analyses (using baseline data), we used generalized linear models with a gamma distribution and an identity link function because residuals from ordinary least squares linear regression models were heteroskedastic and highly skewed. A gamma distribution requires all values to be greater than zero, so 1 minute was added to all participants’ baseline walking minutes-for the cross-sectional models. Akaike Information Criteria (AIC) values were used to compare model fits for a Gaussian (normal) distribution (with an identity link) and Gamma distributions (with identity, inverse, or log links). There were substantial reductions in AIC values between the Gaussian compared to the Gamma distributions, but minimal change between the three different linking functions. Thus, we used Gamma distributions with identity links to improve interpretability and comparability with the longitudinal analyses. We also assessed for non-linearity of the fully adjusted associations by including squared and cubed terms of the built environment predictors, and then testing if model fit was improved using likelihood ratio tests.

For the longitudinal analyses, we used fixed-effects linear regression models to estimate associations for within-person change in walkability and specific built characteristics (population counts, diversity of destinations, and street connectivity) with changes in walking. We created change scores by subtracting follow-up measurements of built environment characteristics from baseline measurements. Because fixed-effects models control for measured and unmeasured confounding by time-invariant covariates [15], only time-varying covariates were included in the models [42]. Linear regression models were used to estimate beta coefficients (*b*) and 95% Confidence Intervals (CI) for changes in built environment characteristics and walking minutes, while adjusting for time-varying covariates. We also assessed for non-linearity of the fully adjusted associations by including squared and cubed terms of the built environment predictors, and then testing if model fit was improved using partial F tests.

Effect modification of the associations between the built environment and walking was examined by including interaction (product) terms for education and household low-income status with the built environment measure separately in each model. Models with significant interaction terms were stratified by the SES indicator and adjusted for the time-varying covariates.

Sensitivity analyses were conducted to account for baseline walking in the longitudinal associations and to test the two alternative measures of household income as potential effect modifiers (in both the cross-sectional and longitudinal analyses). Listwise deletion was used for missing data. Analyses were conducted using R version 4.0.3, and the level of significance was set at *P* < 0.05 for all analyses.

## Results

### Comparison of characteristics between movers and non-movers

There were statistically significant differences (*P* < 0.05) between movers (*n* = 703) and non-movers (*n* = 8094) at baseline. Non-movers were older (mean 55 versus 53 years of age) and more likely to be married/common law (78% versus 69%) compared to movers (Supplementary Material).

### Sample characteristics (movers only)

The majority of participants were women (64%), and at baseline most participants were married/common law (69%), with participant ages ranging from 37 to 77 years (mean = 53, SD (Standard Deviation) = 9 years; Table 1). Most participants completed at least some post-secondary education (79%), and most were from a household above the Alberta median household income cut point (71%). Most participants reported some walking at baseline (79%) and follow-up (84%), with a mean increase from 210 to 271 minutes/week at follow up (SD =381). The time between completing the surveys ranged from 0 to 7 years (mean = 2.9, SD = 1.4 years). Seventy percent of participants completed the follow-up survey in a different season than their baseline survey.

**Table 1:** Sociodemographic characteristics of the cohort (*n* = 703), Alberta’s Tomorrow Project, Canada, 2008–2015

	Overall (*n* = 703)	Decreased Walkability (*n* = 235)	Minimal Change (*n* = 234)	Increased Walkability (*n* = 234)	P*
**Demographics at baseline**					
Age, in years (mean (SD))	52.54 (9.14)	53.35 (8.63)	51.94 (9.47)	52.33 (9.29)	0.227
Sex – women (%)	452 (64.3)	150 (63.8)	153 (65.4)	149 (63.7)	0.913
Low-income household (%)^a^	204 (29.0)	79 (33.6)	67 (28.6)	58 (24.7)	0.107
High school or less (%)	149 (21.2)	60 (25.5)	36 (15.4)	53 (22.6)	0.022
Relationship status (%)					0.986
Married/common Law	486 (69.1)	160 (68.1)	167 (71.4)	159 (67.9)	
Single/never Married	41 (5.8)	14 (6.0)	12 (5.1)	15 (6.4)	
Separated/divorced	142 (20.2)	49 (20.9)	45 (19.2)	48 (20.5)	
Widowed	34 (4.8)	12 (5.1)	10 (4.3)	12 (5.1)	
Children in the home – at least one (%)	216 (30.7)	61 (26.0)	80 (34.2)	75 (32.1)	0.134
Household size (mean (SD))	2.61 (1.36)	2.46 (1.22)	2.68 (1.32)	2.67 (1.52)	0.145
**Walking**					
Baseline walking - minutes per week (mean (SD))	209.87 (256.52)	215.60 (246.45)	205.50 (260.98)	208.48 (262.84)	0.909
Change in walking minutes per week (mean (SD))	61.09 (381.06)	50.15 (374.43)	60.03 (381.65)	73.15 (388.29)	0.807
**Change in covariates**					
No change in relationship status (%)	611 (86.9)	207 (88.1)	202 (86.3)	202 (86.3)	0.759
Same season (%)	210 (29.9)	68 (28.9)	66 (28.2)	76 (32.5)	0.558
Years between surveys (mean (SD))	2.94 (1.39)	2.94 (1.45)	2.97 (1.44)	2.91 (1.27)	0.897

*P values for ANOVAs or chi-square tests, comparing the three walkability-change groups

^a^Household low-income status was determined using the 2006 median income level for Alberta, estimated from the 2006 Statistics Canada Census

### Neighborhood built environments

Participants’ mean baseline neighborhood walkability value was 57.02 (SD = 37.69; Table 2). On average, participants moved to neighborhoods with − 0.25 points lower walkability (SD = 47.91). At baseline, participants’ mean connectivity scores were 15.60 (SD = 9.50) and mean change in connectivity was 1.06 (SD = 10.88). Participants lived in neighborhoods with a mean of 4.75 different destination types (SD = 6.03) at baseline and moved to areas with fewer destination types (mean = − 0.32, SD = 7.81) compared to their previous neighborhood. Mean population counts at baseline were 1054 (SD = 692), and participants moved to areas with lower population counts (mean = − 17, SD = 883), on average, relative to their baseline neighborhoods.

**Table 2:** Built environment characteristics of the cohort (*n* = 703), Alberta’s Tomorrow Project, Canada, 2008–2015

		Highest education level attained		Household income^a^	
	Overall (*n* = 703)	High school or less (*n* = 149)	> High school (*n* = 554)	Low-income (*n* = 204)	Not low-income (*n* = 499)
**Walkability**					
Baseline walkability (mean (SD))	57.02 (37.69)	58.00 (38.56)	56.75 (37.48)	65.53 (36.07)	53.54 (37.81)
Change in walkability (mean (SD))	−0.25 (47.91)	−1.56 (52.06)	0.11 (46.78)	−2.05 (45.14)	0.49 (49.03)
Walkability change tertiles					
Decreased walkability (%)	235 (33.4)	60 (40.3)	175 (31.6)	79 (38.7)	156 (31.3)
Minimal change (%)	234 (33.3)	36 (24.2)	198 (35.7)	67 (32.8)	167 (33.5)
Increased walkability (%)	234 (33.3)	53 (35.6)	181 (32.7)	58 (28.4)	176 (35.3)
**Specific built characteristics**					
Baseline population count (mean (SD))	1054.22 (691.57)	935.24 (532.93)	1086.22 (725.45)	1160.86 (693.29)	1010.62 (686.80)
Change in population count (mean (SD))	−17.31 (883.18)	51.69 (905.20)	−35.87 (877.06)	−19.69 (880.51)	− 16.34 (885.14)
Baseline number of destination types (mean (SD))	4.75 (6.03)	5.19 (6.57)	4.63 (5.87)	5.43 (5.75)	4.47 (6.12)
Change in number of destination types (mean (SD))	−0.32 (7.81)	−0.54 (8.61)	−0.27 (7.59)	−0.21 (7.66)	− 0.37 (7.87)
Baseline connectivity (mean (SD))	15.60 (9.50)	15.97 (10.09)	15.50 (9.34)	18.31 (10.81)	14.50 (8.68)
Change in connectivity (mean (SD))	1.06 (10.88)	0.10 (10.75)	1.31 (10.91)	−0.42 (10.93)	1.66 (10.81)

^a^Household low-income status was determined using the 2006 median income level for Alberta, estimated from the 2006 Statistics Canada Census

### Cross-sectional associations between built environment characteristics and walking at baseline

Neighborhood street connectivity was negatively associated with minutes walked per week at baseline (*b* = − 1.75, 95% CI: − 3.26, − 0.24). In contrast, neighborhood population count was positively associated with minutes walked per week at baseline (*b* = 0.03, 95% CI: 0.01, 0.07). Neighborhood walkability and diversity of destinations were not significantly associated with baseline walking in the overall sample. There was no evidence of non-linear associations between the built environment variables and walking. However, there was evidence of confounding, with several of the estimated association changing substantially after covariate adjustment (crude and adjusted estimates shown in Table 3). Sensitivity analyses testing for effect modification by other household income measures (income tertiles and low-income line threshold) produced similar results (Supplementary Material).

**Table 3:** Cross-sectional associations between built characteristics and walking, Alberta’s Tomorrow Project, Canada (*n* = 703), 2008–2015

	Overall sample	Overall sample			Highest education level attained		Household income^a^	
	Crude (unadjusted) (*n* = 703)	Adjusted (*n* = 703)	*P*-value for interaction term		High school or less (*n* = 149)	> High school (*n* = 554)	Low-income (*n* = 204)	Not low-income (*n* = 499)
	*b* (95% CI)	*b* (95% CI)	education income		*b* (95% CI)	*b* (95% CI)	*b* (95% CI)	*b* (95% CI)
**Built environment characteristic**								
Walkability (per 10-unit increase)	15.52 (−1.51, 34.68)	7.27 (− 11.73, 28.97)	0.069	0.081	− 20.61 (− 55.72, 14.50)	17.52 (− 4.44, 39.48)	−17.01 (− 49.28, 15.25)	17.56 (− 5.40, 40.52)
Connectivity	−0.63 (− 2.55, 1.83)	−1.75* (− 3.26, − 0.24)	**0.029**	**0.016**	−3.74* (− 5.42, − 2.06)	**− 0.73 (− 2.77, 1.32)**	−3.47* (− 4.87, − 2.07)	**−0.03 (− 2.50, 2.43)**
Diversity of destinations	2.99 (− 0.37, 6.91)	1.16 (− 2.43, 5.32)	0.063	0.076	−3.80 (− 9.51, 1.90)	2.84 (− 1.28, 6.97)	−3.50 (− 8.86, 1.86)	2.57 (− 1.60, 6.74)
Population count	0.04* (0.01, 0.07)	0.03* (0.01, 0.07)	0.226	0.281				

Note. The cross-sectional analyses were conducted using baseline data. Each model included only one of the built environment variables. Overall adjusted models included age, sex, relationship status, presence/absence of children at home, and season of survey completion. Models with stratum-specific estimates were adjusted for the same covariates and were included in the table wherever the *P* value for the interaction term was less than 0.10 (model estimates from models with statistically significant interaction terms (*P* < 0.05) were bolded). Models testing for effect modification by baseline education and household income were each run separately

^a^Household low-income status was determined using the 2006 median income level for Alberta, estimated from the 2006 Statistics Canada Census

*P < 0.05

Only the statistical interaction terms for education and household income with street connectivity were statistically significant (Table 3). The associations between street connectivity and baseline walking were only significant among the lower SES groups (lower education: *b* = − 3.74, 95% CI: − 5.42, − 2.06; lower income: *b* = − 3.47, 95% CI: − 4.87, − 2.07). Statistical interactions for walkability and diversity of destinations with education and income approached statistical significance (*P*s < 0.09*).* Although none of the stratified estimates were statistically significant, for both these sets of associations, the coefficient point estimates were negative amongst the lower SES groups and positive amongst the higher SES groups (Table 3).

### Longitudinal associations between changes in built environment characteristics and walking following neighborhood relocation

There were no statistically significant associations between changes in the built environment characteristics and walking for the overall sample (Tables 4 and 5). There was also no evidence of non-linear associations between the built environment variables and walking. There was limited evidence of confounding, with few of the estimates changing substantially after covariate adjustment (crude and adjusted estimates shown in Tables 4 and 5).

**Table 4:** Longitudinal associations between walkability and walking, Alberta’s Tomorrow Project, Canada (*n* = 703), 2008–2015

	Overall sample	Overall sample			Highest education level attained		Household income^a^	
	Crude (unadjusted) (*n* = 703)	Adjusted (*n* = 703)	*P*-value for interaction term		High school or less (*n* = 149)	> High school (*n* = 554)	Low-income (*n* = 204)	Not low-income (*n* = 499)
	*b* (95% CI)	*b* (95% CI)	education income		*b* (95% CI)	*b* (95% CI)	*b* (95% CI)	*b* (95% CI)
**Change in built environment characteristics (baseline to follow up)**								
Walkability (per 10-unit increase)	0.44 (− 20.21, 21.08)	0.41 (− 20.02, 20.84)	0.076	0.502	− 31.08 (− 71.78, 9.63)	11.52 (− 11.87, 34.90)		
% Walkability	0.003* (0.00, 0.01)	0.003 (0.00, 0.01)	**0.030**	0.487	0.01* (0.00, 0.01)	**0.00 (− 0.00, 0.00)**		
Walkability-change tertiles (REF = minimal change)								
Decreased walkability	−9.89 (− 79.06, 59.29)	−9.03 (− 77.48, 59.41)	0.785	0.772				
Increased walkability	13.11 (− 56.13, 82. 36)	12.60 (− 56.12, 81.31)	0.666	0.865				

Note. Each model included only one of the built environment variables. Overall adjusted models included relationship status, children at home, season of survey completion, follow-up survey type, and years between surveys, as time-varying coefficients. Models with stratum-specific estimates were adjusted for the same covariates and were included in the table wherever the *P* value for the interaction term was less than 0.10 (model estimates from models with statistically significant interaction terms (*P* < 0.05) were bolded). Models testing for effect modification by baseline education and household low-income status were each run separately

^a^Household low-income status was determined using the 2006 median income level for Alberta, estimated from the 2006 Statistics Canada Census

*P < 0.05

**Table 5:** Longitudinal associations between specific built characteristics and walking, Alberta’s Tomorrow Project, Canada, 2008–2015

	Overall sample	Overall sample			Highest education level attained		Household income^a^	
	Crude (unadjusted) (*n* = 703)	Adjusted (*n* = 703)	*p*-value for interaction term		High school or less (*n* = 149)	> High school (*n* = 554)	Low-income (*n* = 204)	Not low-income (*n* = 499)
	*b* (95% CI)	*b* (95% CI)	education income		*b* (95% CI)	*b* (95% CI)	*b* (95% CI)	*b* (95% CI)
**Change in built environment (baseline to follow up)**								
Connectivity	−0.51 (− 3.10, 2.09)	−0.18 (− 2.77, 2.40)	0.564	0.231				
Diversity of destinations	−0.20 (− 3.82, 3.42)	−0.46 (− 4.05, 3.12)	**0.036**	0.604	**−6.90 (− 13.94, 0.14)**	**1.85 (− 2.27, 5.96)**		
Population count	0.01 (− 0.03, 0.04)	0.01 (− 0.02, 0.04)	0.759	0.882				

Note. Each model included only one of the built environment variables. Overall adjusted models included relationship status, children at home, season of survey completion, follow-up survey type, and years between surveys, as time-varying coefficients. Models with stratum-specific estimates were adjusted for the same covariates and were included in the table wherever the P value for the interaction term was less than 0.10 (model estimates from models with statistically significant interaction terms (*P* < 0.05) were bolded. Models testing for effect modification by baseline education and household low-income status were each run separately

^a^Household low-income status was determined using the 2006 median income level for Alberta, estimated from the 2006 Statistics Canada Census

*P < 0.05

Among the longitudinal associations, the interaction terms between education and percent change in walkability (Table 4) and education and change in diversity of destinations (Table 5) were significantly associated with change in walking minutes. However, in stratified analyses, only the association between percent change in walkability and change in walking minutes among the low education participants was significant (*b* = 0.01, 95% CI: 0.00, 0.01)) (Table 4).

Sensitivity analyses adjusting for baseline walking and testing for effect modification by other household income measures (income tertiles and low-income line threshold) produced similar results (Supplementary Material).

## Discussion

Our study examined cross-sectional and longitudinal associations between the neighborhood built environment and walking among adults and if individual-level SES modified these associations. Among the cross-sectional analyses, in the overall sample, street connectivity was negatively associated with minutes walked per week, and population count was positively associated with weekly minutes of walking. Walkability and diversity of destinations were not significantly associated with baseline walking for the overall sample. Our results for street connectivity, walkability, and diversity of destinations are inconsistent with prior evidence demonstrating positive associations between these built characteristics and walking [43, 44]. We detected evidence of effect modification of cross-sectional associations by SES only for street connectivity. Among the longitudinal associations, there were no statistically significant associations between changes in neighborhood walkability nor the specific built environment characteristics with walking duration for the overall sample. Evidence of effect modification by SES of longitudinal associations was only found for relative walkability-change and change in diversity of destinations by education.

Although our study found statistically significant associations for the overall sample in the cross-sectional analyses, we did not find the same associations for the longitudinal analyses. Our results are similar to those of Braun and colleagues [45] who undertook a similar cross-sectional and longitudinal analysis of the built environment, but with cardiometabolic risk factors. Their study also found mainly non-significant results, with different metabolic risk factors associated with the built environment at baseline (diastolic blood pressure) compared to across time (triglycerides and systolic blood pressure) [45].

Our lack of statistically significant longitudinal findings contrast with those of Hirsch and colleagues [13] who also used a residential relocation study design and fixed effects models, but found positive associations between changes in absolute walkability and walking. However, in contrast to our study that included an overall measure of walking, Hirsch and colleagues [13] examined walking for transportation and leisure as separate outcomes, and found that associations were only statistically significant for transportation walking. Indeed, our results align with the findings of another residential relocation study (that also used fixed effects models) and combined transportation and leisure walking into a single outcome and found no statistically significant associations between changes in walkability and changes in participation or duration of walking [45]. Most of our study participants completed the IPAQ-short version, rather than the IPAQ-long, which prevented separate assessments of transportation and leisure walking.

Our longitudinal findings regarding specific built characteristics mirror some of the mixed results that have been previously found [15, 16]. Changes in neighborhood population count, diversity of destinations, and street connectivity following residential relocation were not related to changes in walking. Although a UK residential relocation study found a positive association for residential density with daily steps [16], an Australian study found a non-statistically significant association between residential density and transportation walking [15]. These two studies also found positive associations between land use mix and walking [15, 16], whereas our study found no relationship, among the overall sample, between increases in diversity of destinations and changes in walking. These differences in findings for specific built characteristics may be attributable to the larger neighborhood buffer sized used in the previous studies (the UK study used a 1 km network buffer [16] and the Australian study used a 1.6 km network buffer [15]). Our neighborhood buffer size of 400 m excluded potential changes in diversity of destinations located outside the buffer that may have still been within walking distance.

Our results contribute to the inconsistent findings regarding differing impacts of the built environment on physical activity for people from different SES groups [19, 20, 46]. A systematic review of longitudinal studies investigating the associations between the built environment and physical activity found little support for effect modification by SES [19]. In a UK residential relocation study, Clary et al. [16] tested for effect modification by SES within their associations between changes in various built environment measures and accelerometer-derived physical activity measures. Their study found evidence of effect modification only for the association between increases in accessibility to public transit (a composite measure of proximity, number of stops, and frequency of service) with daily steps, such that there were no statistically significant associations among the two higher SES groups, but a marginally significant negative association for the low SES group (i.e., defined by social housing recipient status [16]). Our findings are similar to Clary at al [16] because most associations had no evidence of effect modification by SES and we had some preliminary evidence of negative associations among the low SES groups.

The study has several strengths, including the use of both a cross-sectional and a longitudinal design and the testing of modification by SES indicators. This study is relevant to requests for more policy-relevant research methods [47, 48] because we examined the impact of specific built environment characteristics, which may be more useful to policymakers compared to multi-component walkability measures. Furthermore, we estimated built environment characteristics using data from the same year that the participant completed each questionnaire (except for population count data, where the census data were weighted to approximate the years where the census was not collected). Several other residential relocation studies have used WalkScore®, which is a proprietary measure of walkability, and thus those studies only had the most current data available [13, 45].

The study was limited by relatively small subsamples of participants who could be classified as having low SES, which decreased the precision of our estimates and may have reduced our ability to detect statistically significant associations and interactions. The overall ATP cohort has relatively high SES [23] and is likely affected by a “healthy volunteer” bias that is common among prospective cohort studies [49, 50]. However, the ATP cohort has rates of chronic diseases (e.g., diabetes and hypertension) that are similar to the general Canadian population [51]. Our study was also limited by the self-report measures of walking that did not specify the location where walking occurred and did not differentiate by walking purpose. Our use of Euclidean buffers meant that the geographically defined neighborhoods were standardized across participants, however, this delineation also meant that while certain built environment features were present in the buffer they might not have been easily accessible via walking [52]. We did not have access to data regarding participants’ moving date or their reasons for moving, and as such, we were unable to consider exposure time in neighborhood or reasons for moving (e.g., changing occupations or familial responsibilities). Similarly, there was still potential for residential self-selection bias, even in the longitudinal analyses because some adults who were interested in increasing their walking may have chosen to move to a neighborhood with greater walkability. Finally, the study sample consisted of middle-aged adults (ATP recruitment inclusion criteria included adults aged 35–69 years only) who relocated neighborhoods; thus, the results may not be generalizable to younger adults or non-movers.

Given the limited number and mixed findings on differential impacts of the built environment for SES groups, there is a need for more longitudinal research examining equity impacts of changing the built environment. The mixed evidence so far [19, 20, 46], in addition to our own findings, precludes us from making definitive recommendations in regard to how and which urban planning policies should be modified to encourage more walking among low SES adults. Nevertheless, to improve our understanding and to generate evidence that might inform policy in the future, methodological improvements in future studies examining associations between the built environment and walking among low SES adults may be needed. For example, future studies would benefit from context-specific measures of walking and from separating leisure and transportation walking when investigating the impact of changes in the built environment on walking [53, 54]. It would also be useful for future studies to examine built characteristics that may be particularly relevant for adults with low SES (e.g., transit stops, safety, low-cost or free recreation centers). Such studies may involve natural experiments that involve examining the effects of modifying specific features of the built environment on walking among residents of low SES neighbourhoods. Regardless of our findings, other interventions may be needed to increase population levels of physical activity to avoid negatively impacting adults with lower SES. For instance, more fundamental or ‘upstream’ interventions (e.g., living wage policies) may be needed to address socioeconomic disparities in physical activity [55, 56].

## Conclusions

Our findings suggest that an increase in neighborhood walkability, relative to the prior neighborhood, is associated with increases in walking after relocating neighborhoods. Despite street connectivity and population count being associated with walking in the cross-sectional analyses, these built environment variables were not associated with changes in walking following residential relocation. We found limited evidence that these associations differ by education and household income. Although the evidence is weak, our findings also suggest that changes in neighbourhood walkability, following residential relocation, might more strongly affect walking among low SES adults. Further longitudinal research is needed that examines specific built environment characteristics and walking for different purposes among disadvantaged populations.

## Supplementary Information

**Additional file 1: Table S1.** Sociodemographic Characteristics of Movers (the Study Cohort) and Non-Movers (Excluded) at Baseline. **Table S2.** Longitudinal associations between walkability and walking, including baseline walking as a covariate, Alberta’s Tomorrow Project, Canada (*n* = 703), 2008–2015. **Table S3.** Longitudinal associations between specific built characteristics and walking, including baseline walking as a covariate, Alberta’s Tomorrow Project, Canada, 2008–2015. **Table S4.** Cross-sectional associations between built characteristics and walking, with additional income effect modifiers. **Table S5.** Longitudinal associations between walkability and walking, with additional income effect modifiers. **Table S6.** Longitudinal associations between built characteristics and walking, with additional income effect modifiers.

## Data Availability

The data that support the findings of this study are available from Alberta’s Tomorrow Project, but restrictions apply to the availability of these data, which were used under license for the current study, and so are not publicly available. Data are available, however, from the authors upon reasonable request and with permission of Alberta’s Tomorrow Project. More information can be obtained via https://myatpresearch.ca.

## Abbreviations

SES: Socioeconomic status
ATP: Alberta’s Tomorrow Project
CI: Confidence interval
GIS: Geographic Information Systems
IPAQ: International Physical Activity Questionnaire
ANOVA: Analysis of variance
SD: Standard deviation
