# Supplementary material for: Cross-sectional and longitudinal associations between the built environment and walking: effect modification by socioeconomic status
Source: BMC Public Health. 2022 Jun 21;22:1233. doi: 10.1186/s12889-022-13611-0 (PMC9210749; doi:10.1186/s12889-022-13611-0)
Supplement: Supplementary file 1 — Additional file 1: Table S1. Sociodemographic Characteristics of Movers (the Study Cohort) and Non-Movers (Excluded) at Baseline. Table S2. Longitudinal associations between walkability and walking, including baseline walking as a covariate, Alberta’s Tomorrow Project, Canada (n = 703), 2008–2015. Table S3. Longitudinal associations between specific built characteristics and walking, including baseline walking as a covariate, Alberta’s Tomorrow Project, Canada, 2008–2015. Table S4. Cross-sectional associations between built characteristics and walking, with additional income effect modifiers. Table S5. Longitudinal associations between walkability and walking, with additional income effect modifiers. Table S6. Longitudinal associations between built characteristics and walking, with additional income effect modifiers. [file 12889_2022_13611_MOESM1_ESM.docx]

**SUPPLEMENTARY MATERIAL**

*Detailed description of built environment measures*

The neighborhood built environment exposures for this analysis consisted of specific built environment characteristics (population counts, diversity of destinations, and street connectivity) and a composite walkability index estimated for a 400 meter Euclidean buffer around each participant’s residential geo-located 6-digit postal codes (at baseline and follow-up). A 400-meter buffer was chosen because it represents the approximate distance that can be travelled after walking approximately 5-minutes (1). Previous studies estimating associations between the built environment and walking have also used a 400-meter Euclidean buffer to define the neighborhood area (2, 3). Prior research, conducted in Calgary, Alberta, has shown that postal code locations provide a reasonably accurate proxy for urban addresses, with 88% of postal codes being located within 200 meters of the true address (4). Geographic Information Systems (GIS) were used to process built environment data for all urban postal codes in Alberta for each year between 2008 and 2015, and participant home locations were linked to objective built environment data with their 6-digit postal code for baseline and follow-up based on the year they completed each survey.

Population counts were estimated using 2006, 2011, and 2016 Statistics Canada Census data at the dissemination block level. Population counts were estimated based on the geometric overlap of the 400-meter buffers and the census dissemination blocks. The percentage of each buffer overlap was multiplied by the population count of the dissemination block and summed to create a weighted count for the buffer. Population counts for non-census years were estimated based on averages imputed from the available census data. Diversity of destinations was estimated by summing the number of different destination types (classified by Standard Industry Codes obtained from DMTI Spatial’s CanMap Enhanced Points of Interest data). Examples of different destination types include hardware stores, department stores, grocery stores, restaurants, banks, libraries, liquor stores, barbershops, museums, and schools. Street connectivity was estimated by the number of three and four-way street intersections within the buffer (derived from DMTI Spatial’s CanMap Streetfiles and Route Logistics dataset), but with three-way intersections weighted half as strongly as four-way intersections.

For the overall walkability index, specific built environment characteristics were standardized relative to the maximum value of each characteristic observed between 2008 and 2015 across all urban postal codes in Alberta (making the scores relative to walkability index scores across the province). The maximum values found for each built characteristic are: 1) population count = 7956, 2) number of different destination types = 23, 3) number of four-way intersections = 51, and 4) number of three-way intersections = 92. This percent of maximum possible (“POMP”) method (5, 6) results in a standardized score for each built characteristic that is a proportional score bound by 0 and 100%. The four standardized scores were then summed together, with three-way intersections given a weight of 0.5 and the other scores given a weight of one. The equation below shows the calculation of the walkability index, which has a theoretical minimum of 0 and maximum overall score of 350.

$$Walkability Score= \left( 1\times\left( \frac{\# residents}{7956} \right)\times100 \right) + \left( 1\times\left( \frac{\# of different destination types}{23} \right)\times100 \right)+ \left( 1\times\left( \frac{\# of four way intersections}{51} \right)\times100 \right)+ \left( 0.5\times\left( \frac{\# of three way intersections}{92} \right)\times100 \right)$$

A meta-analysis examining components of walkability found that intersection density had the strongest association with walking compared to our other two components (7). Thus, we weighted intersection density higher than population density or diversity of destinations. Similar to other measures of connectivity (8), the three-way intersections variable in our index was given a weight of 0.5 (instead of one) because three-way intersections have been shown to have a weaker association with walking compared to four-way intersections (9).

**References**

1. Ellis G, Hunter R, Tully MA, Donnelly M, Kelleher L, Kee F. Connectivity and physical activity: using footpath networks to measure the walkability of built environments. Environment and Planning B: Planning and Design. 2016;43(1):130-51.

2. James P, Berrigan D, Hart JE, Hipp JA, Hoehner CM, Kerr J, et al. Effects of buffer size and shape on associations between the built environment and energy balance. Health & place. 2014;27:162-70.

3. Hoehner CM, Ramirez LKB, Elliott MB, Handy SL, Brownson RC. Perceived and objective environmental measures and physical activity among urban adults. American journal of preventive medicine. 2005;28(2):105-16.

4. Bow CJD, Waters NM, Faris PD, Seidel JE, Galbraith PD, Knudtson ML, et al. Accuracy of city postal code coordinates as a proxy for location of residence. International journal of health geographics. 2004;3(1):5.

5. Moeller J. A word on standardization in longitudinal studies: don't. Frontiers in psychology. 2015;6:1389.

6. Cohen P, Cohen J, Aiken LS, West SG. The problem of units and the circumstance for POMP. Multivariate behavioral research. 1999;34(3):315-46.

7. Ewing R, Cervero R. Travel and the built environment: a meta-analysis. Journal of the American planning association. 2010;76(3):265-94.

8. Iravani H, Rao V. The effects of New Urbanism on public health. Journal of Urban Design. 2020;25(2):218-35.

9. Herrmann T, Gleckner W, Wasfi RA, Thierry B, Kestens Y, Ross NA. A pan-Canadian measure of active living environments using open data. Health reports. 2019;30(5):16-26.

***Supplementary Analyses: Comparison of Movers and Non-Movers***

| Table S1 – Sociodemographic Characteristics of Movers (the Study Cohort) and Non-Movers (Excluded) at Baseline | | | |
| --- | --- | --- | --- |
|  | Non-movers  (*n* = 8094) | Movers  (*n* = 703) | *P** |
| **Demographics at baseline** |  |  |  |
| Age, in years (mean (SD)) | 55.25 (8.98) | 52.54 (9.14) | <0.001 |
| Sex – women (%) | 4979 (61.5) | 452 (64.3) | 0.157 |
| Low-income household (%)^a^ | 2139 (26.4) | 204 (29.0) | 0.148 |
| High school or less (%) | 1604 (19.8) | 149 (21.2) | 0.408 |
| Relationship status (%) |  |  | <0.001 |
| Married/common Law | 6315 (78.0) | 486 (69.1) |  |
| Single/never Married | 470 (5.8) | 41 (5.8) |  |
| Separated/divorced | 955 (11.8) | 142 (20.2) |  |
| Widowed | 354 (4.4) | 34 (4.8) |  |
| Children in the home – at least one (%) | 2208 (27.3) | 216 (30.7) | 0.055 |
| Household size (mean (SD)) | 2.61 (1.24) | 2.61 (1.36) | 0.951 |
| Baseline walking - minutes per week  (mean (SD)) | 230.04 (264.43) | 209.87 (256.52) | 0.052 |
| **P* values for independent samples t-tests or chi-square tests, comparing the non-movers to the movers.  ^a^Household low-income status was determined using the 2005 median income level for Alberta, estimated from the 2006 Statistics Canada Census. | | | |

***Supplementary Analyses: Including baseline walking as a covariate in the longitudinal models***

| Table S2. Longitudinal associations between walkability and walking, including baseline walking as a covariate, Alberta’s Tomorrow Project, Canada (*n* = 703), 2008-2015 | | | | | | | | | | |
| --- | --- | --- | --- | --- | --- | --- | --- | --- | --- | --- |
|  | Overall sample | Overall sample |  | |  | Highest education level attained | |  | Household income^a^ | |
|  | Crude  (unadjusted)  (*n* = 703) | Adjusted  (*n* = 703) | *P*-value for interaction term | |  | High school  or less  (*n* = 149) | > high  school  (*n* = 554) |  | Low-income  (*n* = 204) | Not low-income  (n =499) |
|  | *b*  (95% CI) | *b*  (95% CI) | education income | |  | *b*  (95% CI) | *b*  (95% CI) |  | *b*  (95% CI) | *b*  (95% CI) |
| **Change in built environment characteristics (baseline to follow up)** |  |  | | | | | | | | |
| Walkability (per 10-unit increase) | 0.44  (-20.21, 21.08) | -1.40  (-19.07 – 16.27) | 0.102 | 0.623 |  |  |  |  |  |  |
| % Walkability | 0.003*  (0.00, 0.01) | 0.002  (-0.00 – 0.00) | **0.010** | 0.279 |  | **0.01***  **(0.00, 0.01)** | **-0.00**  **(-0.00, 0.00)** |  |  |  |
| Walkability-change tertiles  (REF = minimal change) |  |  |  |  |  |  |  |  |  |  |
| Decreased walkability | -9.89  (-79.06, 59.29) | 1.48  (-57.73 – 60.68) | 0.955 | 0.710 |  |  |  |  |  |  |
| Increased walkability | 13.11  (-56.13, 82. 36) | 16.36  (-43.08 – 75.79) | 0.697 | 0.875 |  |  |  |  |  |  |
| Note. Each model included only one of the built environment variables. Overall adjusted models included relationship status, children at home, season of survey completion, follow-up survey type, and years between surveys, as time-varying coefficients, as well as baseline walking. Models with stratum-specific estimates were adjusted for the same covariates and were included in the table wherever the *P* value for the interaction term was less than 0.10 (model estimates from models with statistically significant interaction terms (*P*<0.05) were bolded). Models testing for effect modification by baseline education and household low-income status were each run separately.  ^a^Household low-income status was determined using the 2005 median income level for Alberta, estimated from the 2006 Statistics Canada Census.  **P* <0.05 | | | | | | | | | | |

| Table S3. Longitudinal associations between specific built characteristics and walking, including baseline walking as a covariate, Alberta’s Tomorrow Project, Canada, 2008-2015 | | | | | | | | | | |
| --- | --- | --- | --- | --- | --- | --- | --- | --- | --- | --- |
|  | Overall sample | Overall sample |  | |  | Highest education level attained | |  | Household income^a^ | |
|  | Crude  (unadjusted)  (*n* = 703) | Adjusted  (*n* = 703) | p-value for interaction term | |  | High school  or less  (*n* = 149) | > high  school  (*n* = 554) |  | Low-income  (*n* = 204) | Not low-income  (n =499) |
|  | *b*  (95% CI) | *b*  (95% CI) | education income | |  | *b*  (95% CI) | *b*  (95% CI) |  | *b*  (95% CI) | *b*  (95% CI) |
| **Change in built environment**  **(baseline to follow up)** |  |  | | | | | | | | |
| Connectivity | -0.51  (-3.10, 2.09) | 0.16  (-2.07 – 2.40) | 0.241 | 0.173 |  |  |  |  |  |  |
| Diversity of destinations | -0.20  (-3.82, 3.42) | -0.73  (-3.82 – 2.37) | 0.124 | 0.804 |  |  |  |  |  |  |
| Population count | 0.01  (-0.03, 0.04) | -0.003  (-0.03 – 0.02) | 0.816 | 0.742 |  |  |  |  |  |  |
| Note. Each model included only one of the built environment variables. Overall adjusted models included relationship status, children at home, season of survey completion, follow-up survey type, and years between surveys, as time-varying coefficients, as well as baseline walking. Models with stratum-specific estimates were adjusted for the same covariates and were included in the table wherever the P value for the interaction term was less than 0.10 (model estimates from models with statistically significant interaction terms (P<0.05) were bolded. Models testing for effect modification by baseline education and household low-income status were each run separately.  ^a^Household low-income status was determined using the 2005 median income level for Alberta, estimated from the 2006 Statistics Canada Census.  **P* <0.05 | | | | | | | | | | |

***Supplementary Analyses: Testing two other operational definitions of income as potential effect modifiers***

| Table S4: Cross-sectional associations between built characteristics and walking, with additional income effect modifiers | | | | | | | | | | | | | | | |
| --- | --- | --- | --- | --- | --- | --- | --- | --- | --- | --- | --- | --- | --- | --- | --- |
|  |  |  | | Income 1^a^ | | |  |  | | Income 2^b^ | | |  | Income 3^c^ | |
|  |  | P-value for interaction | | <60K  N=204 | >60K  N=499 | |  | P-value  for interaction | | Below poverty line  (n=24) | Not below poverty line  (n=679) | | P-value for  interaction | Tertile 1, Tertile, 2, and Tertile 3 (REF)  (ns = 221, 256, & 226) | |
| **Built environment**  **characteristic** |  |  | | *b*  (95% CI) | *b*  (95% CI) | |  |  | | *b*  (95% CI) | *b*  (95% CI) | |  | *b*  (95% CI) | *b*  (95% CI) |
|  |  | |  | | |  | | |  | | |  |  |  |  |
| Walkability (per 10-unit increase) |  | 0.081 | | -17.01  (-49.28, 15.25) | 17.56  (-5.40, 40.52) | |  | 0.030 | | -59.92  (-119.47,  -0.37)* | 9.16  (-10.39 – 28.67) | | 0.162  0.899 |  | |
| Connectivity |  | **0.016** | | **-3.47***  **(-4.87, -2.07)** | **-0.03**  **(-2.50, 2.43)** | |  | 0.491 | |  |  | | 0.140  0.898 |  | |
| Diversity of destinations |  | 0.076 | | -3.50  (-8.86, 1.86) | 2.57  (-1.60, 6.74) | |  | < 0.001 | | -15.76  (-24.37,  -7.15)* | 0.96  (-2.45, 4.38) | | 0.311  0.975 |  | |
| Population count |  | 0.281 | |  |  | |  | 0.109 | |  |  | | 0.237  0.658 |  | |
| Note. The cross-sectional analyses were conducted using baseline data. Each model included only one of the built environment variables. Overall adjusted models included age, sex, relationship status, presence/absence of children at home, and season of survey completion. Models with stratum-specific estimates were adjusted for the same covariates and were included in the table wherever the *P* value for the interaction term was less than 0.10 (model estimates from models with statistically significant interaction terms (*P*<0.05) were bolded). Models testing for effect modification by each of the household income variables were run separately.  ^a^Household low-income status was determined using the 2005 median income level for Alberta, estimated from the 2006 Statistics Canada Census.  ^b^Below poverty line status was determined using the Statistics Canada Market Basket Measure for all urban Alberta regions (separate cute points were calculated for each year and household size  ^c^Income tertiles were created using baseline household income for the sample.  **P* <0.05 | | | | | | | | | | | | | | | |

| Table S5: Longitudinal associations between walkability and walking, with additional income effect modifiers | | | | | | | | | | | |
| --- | --- | --- | --- | --- | --- | --- | --- | --- | --- | --- | --- |
|  |  |  | Income 1^a^ | |  |  | Income 2^b^ | |  | Income 3^c^ | |
|  |  | P-value for interaction | <60K  N=204 | >60K  N=499 |  | P-value  for interaction | Below poverty line  (n=24) | Not below poverty line  (n=679) | P-value for  interaction | Tertile 1, Tertile, 2, and Tertile 3 (REF)  (ns = 221, 256, & 226) | |
| **Change in built environment characteristics (baseline to follow up)** |  |  |  |  |  |  |  |  |  |  |  |
|  |  |  | *b*  (95% CI) | *b*  (95% CI) |  |  | *b*  (95% CI) | *b*  (95% CI) |  | *b*  (95% CI) | *b*  (95% CI) |
| Walkability (per 10-unit increase) |  | 0.502 |  |  |  | 0.151 |  |  | 0.425  0.877 |  | |
| % Walkability |  | 0.487 |  |  |  | 0.082 | -0.90  (-1.92, 0.12) | 0.00  (-0.00, 0.01) | 0.754  0.733 |  | |
| Walkability-change tertiles  (REF = minimal change) |  |  |  |  |  |  |  |  |  |  | |
| Decreased walkability |  | 0.772 |  |  |  | 0.112 |  |  | 0.987  0.773 |  | |
| Increased walkability |  | 0.865 |  |  |  | **0.042** | **-407.33 (-819.79, 5.13)** | **26.58**  **(-42.90, 96.07)** | 0.657  0.163 |  | |
| Note. Each model included only one of the built environment variables. Overall adjusted models included relationship status, children at home, and season of survey completion, follow-up survey type, and years between surveys, as time-varying coefficients. Models with stratum-specific estimates were adjusted for the same covariates and were included in the table wherever the *P* value for the interaction term was less than 0.10 (model estimates from models with statistically significant interaction terms (*P*<0.05) were bolded). Models testing for effect modification by each of the income variables were run separately.  ^a^Household low-income status was determined using the 2005 median income level for Alberta, estimated from the 2006 Statistics Canada Census.  ^b^Below poverty line status was determined using the Statistics Canada Market Basket Measure for all urban Alberta regions (separate cute points were calculated for each year and household size  ^c^Income tertiles were created using baseline household income for the sample.  **P* <0.05 | | | | | | | | | | | |

| Table S6: Longitudinal associations between built characteristics and walking, with additional income effect modifiers | | | | | | | | | | | | | | | |  |
| --- | --- | --- | --- | --- | --- | --- | --- | --- | --- | --- | --- | --- | --- | --- | --- | --- |
|  |  |  | | Income 1 | | |  |  | | Income 2 | | |  | Income 3 | | |
|  |  | P-value for interaction | | <60K  N=204 | >60K  N=499 | |  | P-value  for interaction | | Below poverty line  (n=24) | Not below poverty line  (n=679) | | P-value for  interaction | Tertile 1, Tertile, 2, and Tertile 3 (REF)  (ns = 221, 256, & 226) | | |
| **Change in built environment characteristics (baseline to follow up)** |  |  | | *b*  (95% CI) | *b*  (95% CI) | |  |  | | *b*  (95% CI) | *b*  (95% CI) | |  | *b*  (95% CI) | *b*  (95% CI) | |
|  |  | |  | | |  | | |  | | |  |  |  |  |  |
| Connectivity |  | 0.231 | |  |  | |  | 0.821 | |  |  | | 0.925  0.728 |  | | |
| Diversity of destinations |  | 0.604 | |  |  | |  | **0.047** | | **-22.20**  **(-43.95, -0.45)*** | **0.15**  **(-3.47, 3.78)** | | 0.210  0.510 |  | | |
| Population count |  | 0.882 | |  |  | |  | 0.757 | |  |  | | 0.970  0.293 |  | | |
| Note. Each model included only one of the built environment variables. Overall adjusted models included relationship status, children at home, and season of survey completion, follow-up survey type, and years between surveys, as time-varying coefficients. Models with stratum-specific estimates were adjusted for the same covariates and were included in the table wherever the *P* value for the interaction term was less than 0.10 (model estimates from models with statistically significant interaction terms (*P*<0.05) were bolded). Models testing for effect modification by each of the household income variables were run separately.  ^a^Household low-income status was determined using the 2005 median income level for Alberta, estimated from the 2006 Statistics Canada Census.  ^b^Below poverty line status was determined using the Statistics Canada Market Basket Measure for all urban Alberta regions (separate cute points were calculated for each year and household size  ^c^Income tertiles were created using baseline household income for the sample.  **P* <0.05 | | | | | | | | | | | | | | | | |
